# Role of lactoferrin and its derived peptides in metabolic syndrome treatment

**DOI:** 10.3389/fendo.2025.1562653

**Published:** 2025-04-17

**Authors:** Xicui Zong, Yajing Wang, Yuqing Chen, Penghua Fang, Yi Zhang

**Affiliations:** ^1^ Laboratory Training Center, Nanjing University of Chinese Medicine Hanlin College, Taizhou, Jiangsu, China; ^2^ Department of Endocrinology, Clinical Medical College, Yangzhou University, Yangzhou, China; ^3^ School of Life Sciences, Nanjing Normal University, Nanjing, Jiangsu, China; ^4^ The First School of Clinical Medicine, Nanjing University of Chinese Medicine, Nanjing, Jiangsu, China

**Keywords:** lactoferrin, bioactive peptide, metabolic syndrome, insulin resistance, hypotension

## Abstract

The prevalence of metabolic syndrome is increasing globally year by year, which has prompted researchers to actively seek and develop natural biotherapeutics to address this challenge. Lactoferrin (LF), as a multifunctional iron-binding natural transferrin, has garnered significant attention due to its potential role in regulating metabolism and the immune system. Recent studies show lactoferrin may influence lipid metabolism and glucose-insulin balance, and its levels are linked to body measurements. We systematically summarized the phenotypic and genotypic changes of LF in patients with metabolic syndrome, and the effect of exogenous LF on the treatment of metabolic syndrome. We also recapitulate LF can alleviate insulin resistance by inhibiting the NF-κB inflammatory pathway, activating the IRS/PI3K/Akt/Glut signaling pathway, and inhibiting the renin-angiotensin system to reduce the blood pressure, therefore improving the metabolic syndrome. This provides an important theoretical basis for the clinical application of LF in metabolic syndrome.

## 1 Introduction

Metabolic syndrome (MS) is a worldwide healthcare issue of increasing magnitude, with the number of cases projected to reach approximately 2.568 billion by 2040 (1). MS is defined by metabolic abnormalities, including insulin resistance, central obesity, hyperlipidemia, hyperglycemia, and hypertension, and is also critically involved in the pathogenesis of cardiovascular diseases, strokes, and tumors (2). MS is a condition marked by insulin resistance that can lead to type 2 diabetes mellitus (T2DM). It has been well-documented that insulin resistance results in elevated levels of inflammatory factor markers, such as C-reactive protein (CRP) and cytokine interleukin 6 (IL-6) (3, 4), and promotes adverse outcomes of atherothrombosis through an acceleration of the premature atherosclerosis process (5, 6). Although it is commonly believed that obesity induces the onset of insulin resistance, hepatic insulin resistance is an early step in peripheral insulin resistance, so insulin resistance actually precedes the onset of obesity (7). The accumulation of visceral fat, a typical symptom of obesity, leads to the production of adipokines such as leptin (8), lipocalin (9), C1q tumor necrosis factor-related protein 9 (10), chemerin (11), and retinol-binding protein 4 (12), which are involved in a variety of metabolic processes such as glucose uptake, insulin signaling, and fatty acid oxidation, and are highly correlated with T2DM and cardiovascular and microvascular complications are highly relevant (13, 14).

In addition to active intervention and improvement of the patient’s lifestyle, the clinical treatment of MS focuses on individual or combined drug therapy for specific pathologic features to achieve reduction of insulin resistance, restoration of normal blood glucose, improvement of lipid metabolism disorders, and lowering of blood pressure. However, most of these drugs may cause more side effects, such as rimonabant and sibutramine having psychiatric or cardiovascular risks, respectively, and the pancreatic lipase inhibitors orlistat and metformin can cause gastrointestinal adverse effects (15). Although Chinese medicines with fewer side effects represented by polyphenols, polysaccharides, saponins, and alkaloids can also reduce MS symptoms better, there are still fewer clinical studies, insufficient sample size, and difficulty in extracting and identifying bioactive components (16). In view of these many problems, it is urgent to seek and develop novel natural biological drugs to prevent and treat MS.

In recent years, it has been found that LF is closely related to the development of MS and has the potential to treat MS (17, 18). LF was first found in cow’s milk, and human LF, consisting of 710 amino acids, has a molecular weight of about 80 kDa. It is structurally similar to serum transferrin and can bind to ferric ions, and therefore is categorized as a member of the transferrin family. In addition to being present in most milk secretions, LF is also distributed in mucosal secretions and granules of neutrophils. It is now often used as a food additive and pharmaceutical adjuvant, playing the roles of antioxidant, bacterial inhibition, enhancement of drug efficacy and reduction of drug resistance. LF has been found to have the potential to be used as an antioxidant, drug enhancer, and drug mitigator in MS (18). Studies have reported that it is also involved in the regulation of glucose and lipid uptake, improvement of insulin production and signaling, inhibition of adipogenesis, reduction of inflammation, and oxidative stress associated with metabolic syndrome, among other processes.

## 2 Lactoferrin effective in improving metabolic syndrome

Clinical studies have shown a practical correlation between fluctuations in endogenous LF levels and metabolic disorders, and LF may regulate glucose metabolism, insulin homeostasis and lipid metabolism. Lactoferrin levels were significantly reduced in patients with gestational diabetes, which was linked to hyperglycemic indicators and iron homeostasis disorders, and may serve as a biomarker for detecting different stages of gestational diabetes (19). The concentration of LF in the saliva of healthy individuals was about 40% higher than that of patients with decompensated T2DM, and the release of LF from neutrophils was correspondingly reduced in insulin-resistant subjects (20). Lactoferrin could also enhance insulin signaling and inhibit the activity of RB1 and AMPK, promoting fat production in human adipocytes (21). The expression level of the LF gene was significantly lower in obese patients and negatively correlates with the expression level of inflammatory markers, with fasting triglyceride (TG), body mass index (BMI), and fasting glucose, and with plasma high-density lipoprotein cholesterol (HDL-C) levels, and there was also a significant correlation with the risk of hypertension (22). In severely obese patients, LF concentrations were negatively correlated with postprandial lipemia, oxidative stress parameters (e.g., catalase and glutathione peroxidase), and CRP, suggesting that endogenous LF was elevated and subjects had an improved response to fat load (23).

LF and LF receptor gene variants are associated with the prevalence of disorders of glucolipid metabolism. In subjects with altered glucose tolerance, two LF gene polymorphisms (LF rs1126477 and rs1126478) were associated with HDL-C and TG levels (18). Whereas in metabolically healthy obese patients, there was a significant difference in low-density lipoprotein cholesterol (LDL-C) levels between LTF rs1126477 gene variants, and LDL-C levels were significantly different (18), serum LF concentrations were also negatively correlated with HDL-C levels (24). Polymorphisms in the LF receptor gene (LRP1 rs4759277) have also been associated with fasting insulin levels and homeostatic modeling assessment of insulin resistance in patients with metabolic syndrome (25).

Exogenous supplementation of LF could also improve energy metabolism (26, 27). Three months of oral administration of camel LF capsules to pediatric patients with T2DM resulted in a significant increase in insulin expression and a decrease in serum glucose, suggesting a potential hypoglycemic effect of camel LF (28). Subjects supplemented with LF showed a significant reduction in total and visceral fat accumulation, leading to a decrease in body weight and BMI (29) as well as a decrease in intestinal absorption of TG (30).

## 3 Mechanisms of lactoferrin alleviating the metabolic syndrome

### 3.1 Anti-inflammatory effects of lactoferrin improve insulin resistance

**Figure 1:**
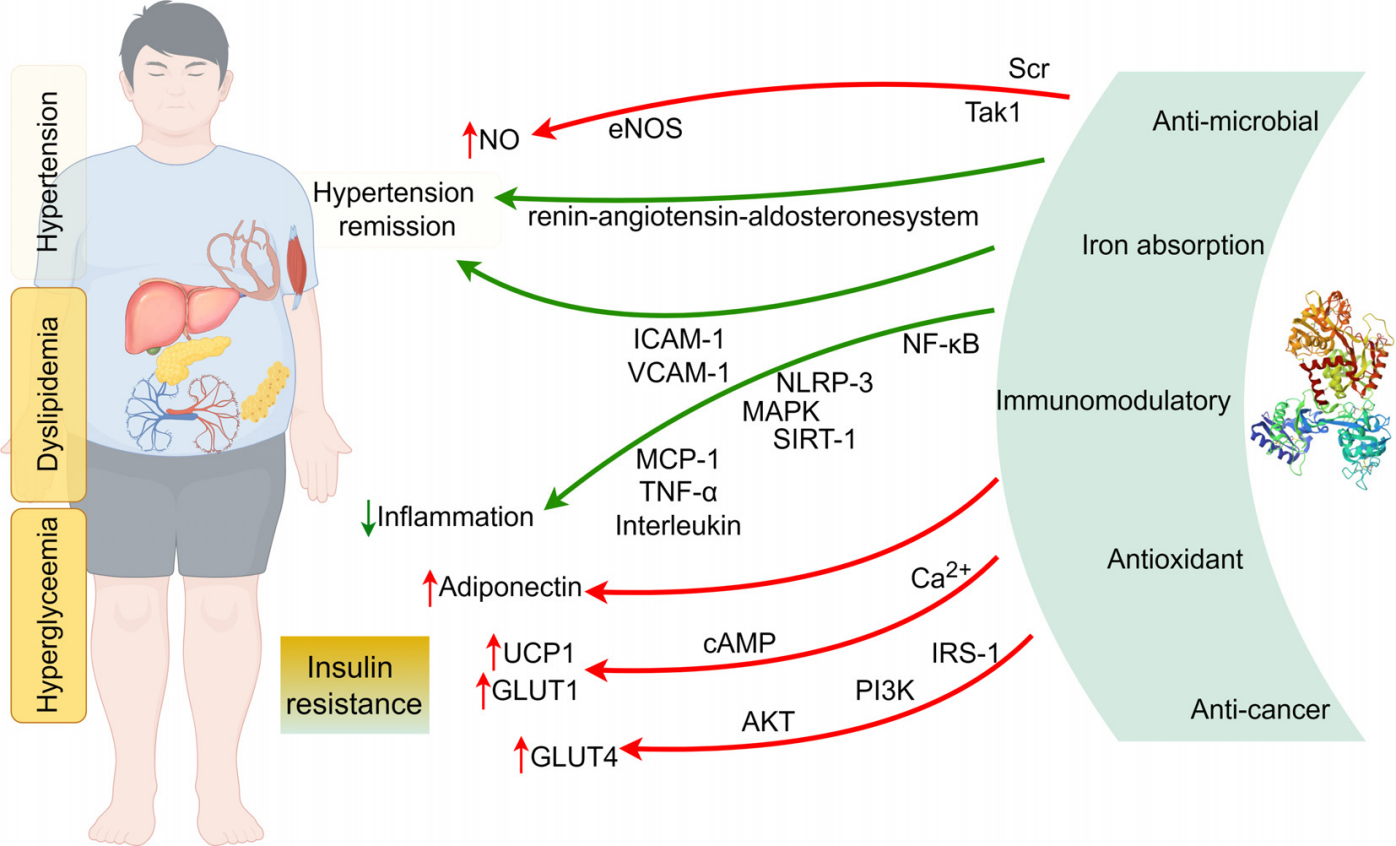
The mechanisms of lactoferrin alleviating the metabolic syndrome (By Figdraw).

Inflammation is an important cause of the development of insulin resistance (31). LF may significantly affect insulin signaling and related functions by reducing inflammation (26). Animal experiments have shown that LF improves the behavioral manifestation of pain in rats with chronic compression injury models and inhibits inflammatory responses by down-regulating the levels of inflammatory cytokines IL-6 and tumor necrosis factor-α (TNF-α), thus exerting analgesic effects (**Figure 1**) (32). Down-regulation of TNF-α and IL-6 mRNA expression in the pancreas of diabetic mice modulates pancreatic inflammatory state to improve pancreatic dysfunction (30). Diabetic LF knockout mice are more susceptible to periodontal disease with increased secretion of pro-inflammatory cytokines compared to diabetic wild-type mice (33). LF inhibits the release of IL-1β in the liver (34), suppresses the expression of monocytes chemochemin-1 (MCP-1) in the liver and adipose tissue of epididymis in obese mice (35), decreases the levels of intercellular cell adhesion molecule-1 (ICAM-1) and vascular cell adhesion molecule-1 (VCAM-1) in mice fed a high-fat diet (22), and reduces the expression modulate the lipopolysaccharide (LPS)-mediated inflammatory cascade (36), mainly by inhibiting LPS-induced secretion of IL-6 by human monocyte cell lines (37), down-regulating LPS-stimulated secretion of IL-10 by macrophages (38), and inhibiting the expression of pro-inflammatory cytokines including TNF-α, IL-1, IL-6 and IL-8 (39), and upregulates lipocalin expression (18).

In T2DM mice, LF ameliorates pancreatic dysfunction by reducing inflammatory responses through regulating the PI3K/AKT signaling pathway. LF reduces serum glycated protein and fasting insulin concentrations and improves hepatic insulin sensitivity (30), and also reduces serum or hepatic levels of TNF-α, IL-6, and IL-1β, reversing abnormal inflammatory responses in diabetic mice (17). In addition, LF can maintain intestinal barrier integrity and alleviate LPS-induced inflammatory responses by attenuating the NF-κB/MAPK pathway (40), and regulate the expression of cytokines, such as TNF-α, IL-6, and IL-1, to exert the protective effect of the intestinal immune barrier (41), rebalance the disorders of glucose-lipid metabolism, and restore inflammatory parameters (42). The effect of lactoferricin bovine (LfcinB) in rats with enteritis led to a decrease in the mRNA expression of pro-inflammatory factors IL-6, IL-1β, and TNF-α in colonic tissues, which mainly inhibited the occurrence and development of inflammation through the NF-κB/NLRP3 signaling pathway and thus achieved the protection of the intestinal mucosal barrier function. MT10, the main product produced after gastric digestion, can prevent inflammatory damage of intestinal organoids by TNF-α and maintain stable growth of intestinal organoid cells (43).

The role of LF as an anti-inflammatory agent has also been validated in *in vitro* cellular-level experiments. In studies on the human hepatocellular carcinoma cell line HepG2 as well as the undifferentiated and pre-differentiated fibroblastic mouse cell line 3T3-L1 under non-inflammatory and inflammatory conditions, it was found that the hypoglycemic activity of LF may be related to the improvement of insulin resistance by regulating the expression of glycoprotein genes and thus exerting the anti-inflammatory mechanism of its activity (44, 45). LF down-regulated the expression of transforming growth factor-β-activated kinase 1 and IL-18, restored the level of AKT (Ser 473) phosphorylation in 3T3-L1 cells, and reduced the expression levels of IL-8, IL-6 and MCP-1 genes in subcutaneous and visceral adipocytes (22).

LF exerts insulin-sensitizing and anti-inflammatory effects by inhibiting the TLR-4/NF-kB/SIRT-1 signaling cascade and correspondingly decreases the expression of serum pro-inflammatory cytokines IL-1β, IL-6, lipocalin 2, and TNF-α, thereby reducing diabetes-related inflammation (28). It directly promotes glucose transport to small intestinal epithelial cells via sodium-dependent glucose transporter 1 through down-regulation of Ca^2+^ and cAMP signaling pathways (46) and leads to increased energy expenditure by promoting uncoupling protein 1 gene expression in brown adipocytes through the cAMP-PKA signaling pathway (47).

### 3.2 Lactoferrin activates IRS-1/PI3K/AKT to improve insulin resistance

Studies have shown that LF upregulates insulin receptor (IR), insulin receptor substrate-1 (IRS-1), glucose transporter 4 (GLUT4), PI3K and AKT in liver protein expression (30), increases peroxisome proliferator-activated receptor γ and regulatory protein SIRT-1 expression (28), and is negatively correlated with chronic inflammation-induced metabolic disorders of insulin resistance, hyperglycemia, and obesity, and positively correlated with insulin sensitivity (48). Huang (49) observed that the PI3K/AKT pathway was blocked in the T2DM state. Lactoferrin can activate the IRS-1/PI3K/AKT pathway by facilitating insulin binding to IR. AKT activation leads to phosphorylation of AS160, which contributes to the translocation of GLUT4 from intracellular vesicles to the cell membrane, thereby improving glucose uptake (30, 34). In addition, the protective effects of LF can be realized through its ability to bind glucose and its anti-inflammatory activity (50). During differentiation of HepG2 and 3T3-L1 cells, lactoferrin increases insulin-induced phosphorylation of AKT (Ser 473), leading to an increase in AMPK (pThr 172) and a decrease in adipogenesis (51). The LF effect of p-AKT has also been found in other diseases, with Alzheimer’s disease patients having reduced levels of PI3K and p-AKT in peripheral blood lymphocyte solution, and significant improvements in all of these metrics with LF (52).

The bioactive peptides that were obtained through modification and alteration were also more successful in mitigating the effect of insulin resistance. The suggested peptide, which has the sequence RER-EtBn, has the ability to stimulate the phosphorylation of its major target, AKT serine, inhibit the phosphorylation of Gsk-3β, and then promote the translocation of the GLUT4 protein to the cell membrane’s surface to promote glucose translocation, all of which have a positive impact on the state of insulin-resistant glucose metabolism (53).

It is evident that LF ameliorates hepatic insulin resistance and pancreatic dysfunction in T2DM mice by regulating the PI3K/AKT signaling pathway. In addition, it has been shown that whey protein can stimulate the translocation of GLUT4 to the plasma membrane of muscle tissue independently of insulin secretion (54), while LF itself can reverse the GLUT4 downregulation triggered by a high-fat diet (30). This may be another potential hypoglycemic mode of action of LF, and its specific mechanism needs to be further investigated.

### 3.3 Lactoferrin inhibits the renin-angiotensin system to regulate blood pressure

Hypertension, as a chronic disease, is the causative agent of a wide range of clinical disorders and often requires long-term medication. The regulatory effects of LF on blood pressure have also received attention, and its antihypertensive effects may be exerted by affecting nitric oxide (NO) synthesis and endothelium-dependent vasodilation. LF treatment significantly down-regulated the high-salt and high-fat-induced renal NLRP3 inflammatory vesicles and protein expression levels of inflammatory factors and regulated the expression levels of mRNAs related to the renin-angiotensin-aldosterone system pathway, which can prevent 8% NaCl diet-induced hypertension and renal injury in mice (55). LF reduces systolic blood pressure, serum adhesion molecules (ICAM-1 and VCAM-1) and aortic reactive oxygen species levels, and improves the endothelium-dependent diastolic function in mice fed a high-fat diet. In addition, LF down-regulated the Tak1/IL-18/eNOS pathway between perivascular adipose tissue and the aorta and promoted NO production in high-fat diet mice, which in turn ameliorated hypertension (22). Dexamethasone-induced systolic blood pressure elevation was lessened by LF administration (56), which also boosted NO generation in bovine aortic endothelial cells (57) and phosphorylated more eNOS in human aortic endothelial cells via a Scr/Akt/eNOS-dependent pathway (22).

Hypotensive peptides derived from lactoferrin have also been identified, and the angiotensin-converting enzyme-inhibiting tripeptide low-density lipoprotein receptor related protein (LRP) derived from bovine lactoferrin, has antihypertensive effects (58). RPYL, identified from the lactoferrin B-derived peptide LfcinB20-25 (RRWQWR), has antihypertensive activity comparable to valsartan (59). The antihypertensive effects of the heptapeptides found in lactoferrin pepsin LF hydrolysate and yeast protein hydrolysate (DPYKLRP) were observed in spontaneously hypertensive rats. The antihypertensive effects were comparable in magnitude and duration to those of the antihypertensive medication captopril (60). Long-term oral treatment of spontaneously hypertensive rats resulted in a considerable reduction in systolic blood pressure as well as a decrease in serum levels of aldosterone, angiotensin II, and the enzyme angiotensin converting enzyme; however, it had no antihypertensive impact on normotensive rats (61). In addition, data suggests that LfcinB20-25, LfcinB17-31, and LfcinB17-22 have a 10-fold *in vitro* antihypertensive effect. RPYL and LIWKL have similar inhibitory effects on angiotensin converting enzyme (ACE)-dependent vasoconstriction. LF hydrolysates, The antihypertensive effects of LfcinB20-25, RPYL and LIWKL may be due to ACE inhibition and induced reduction of vascular tone *in vivo*. The above ex vivo experiments showed that LF-derived peptides have higher ACE inhibitory capacity in ex vivo (62). It has been shown that LF-derived peptides’ mechanisms of hypotensive action involve not only the inhibition of ACE but also interactions with the renin-angiotensin system, the endothelin system, and regulation of gene expression encoding proteins involved in the NO pathway and prostaglandin synthesis (61).

## 4 Conclusions

In summary, the mechanism of LF in human metabolism involves multiple processes, including regulation of glucose and lipid uptake, improvement of insulin production and signal transduction, inhibition of adipogenesis, elevation of HDL cholesterol and reduction of oxidized LDL cholesterol forms, and reduction of inflammation and oxidative stress associated with the metabolic syndrome. Therefore, LF may be an effective therapeutic target for metabolic disorders and is significant for the study of the occurrence and development of various diseases. Simultaneously, LF is a nutritional additive that has received approval from regulatory agencies, with no significant potential side effects identified. Its safety has been corroborated through studies for the treatment of other conditions, including iron-deficiency anemia (63–65). However, LF is in reality degraded in the gastrointestinal tract, so its biological effects may derive mainly from its digestion products rather than from the intact LF molecule. It has been shown that exogenous LF is hydrolyzed by proteases and mainly exists in the form of peptides, which have small molecular masses, are well digested and absorbed, and even exhibit higher biological activities (66). Therefore, further investigation is needed to find out whether LF autocrine is consistent with the effect of exogenously added LF.
